# Probiotics and nanoparticle-mediated nutrient delivery in the management of transfusion-supported diseases

**DOI:** 10.3389/fcimb.2025.1575798

**Published:** 2025-04-11

**Authors:** Wendao Han, Nating Xiong, Lingmei Huang

**Affiliations:** Department of Blood Transfusion, Meizhou People’s Hospital, Meizhou Academy of Medical Sciences, Meizhou, China

**Keywords:** bone marrow, disorders, nanoparticles, nanotechnology, nutraceuticals

## Abstract

Bone marrow is vital for hematopoiesis, producing blood cells essential for oxygen transport, immune defense, and clotting. However, disorders like leukemia, lymphoma, aplastic anemia, and myelodysplastic syndromes can severely disrupt its function, leading to life-threatening complications. Traditional treatments, including chemotherapy and stem cell transplants, have significantly improved patient outcomes but are often associated with severe side effects and limitations, necessitating the exploration of safer, more targeted therapeutic strategies. Nanotechnology has emerged as a promising approach for addressing these challenges, particularly in the delivery of nutraceuticals—bioactive compounds derived from food sources with potential therapeutic benefits. Despite their promise, nutraceuticals often face clinical limitations due to poor bioavailability, instability, and inefficient delivery to target sites. Nanoparticles offer a viable solution by enhancing the stability, absorption, and targeted transport of nutraceuticals to bone marrow while minimizing systemic side effects. This study explores a range of bone marrow disorders, conventional treatment modalities, and the potential of nanoparticles to enhance nutraceutical-based therapies. By improving targeted delivery and therapeutic efficacy, nanoparticles could revolutionize bone marrow disease management, providing patients with more effective and less invasive treatment options. These advancements represent a significant step toward safer and more efficient therapeutic approaches, ultimately improving patient prognosis and overall health.

## Introduction

The emergence of nanotechnology has significantly transformed the treatment and healing processes related to bone diseases, introducing innovative strategies that exceed traditional medical practices (Zhang et al., 2024b). Therapies that incorporate nanomaterials are becoming essential solutions in orthopaedic medicine, utilizing the distinctive characteristics of nanoscale materials to facilitate bone repair and regeneration and also the therapeutic benefits of probiotics (Malmir et al., 2021; Zhang et al., 2021). The nanoscale dimensions of nanoparticles enhance their bioavailability within biological systems, significantly improving their efficacy in medical applications. The rapid advancements in nanotechnological biomaterials are meeting the increasing demand for functional bone grafts and implants, providing innovative and effective solutions to bone regeneration challenges (Wu et al., 2022). In the multidisciplinary domain of tissue engineering, which combines material science, and life sciences, there is a growing focus on the development of nanomaterials, including nanoparticles, nanofibers, and nanocomposites (Fathi-Achachelouei et al., 2019). These materials are being developed to address a variety of bone disorders, including osteoporosis, fractures, and bone defects. By integrating these nanomaterials into biomaterials and drug delivery systems, the goal is to enhance bone growth, increase bone strength, and facilitate faster healing (Bharadwaz and Jayasuriya, 2020). Their ability to accurately target specific cells and tissues significantly improves their therapeutic effectiveness while minimizing potential side effects (Sun et al., 2023b).Probiotics are live microorganisms that provide health advantages to the host when consumed in sufficient quantities. While they have traditionally been linked to gastrointestinal health, recent studies are investigating their potential effects on bone health (Uusitupa et al., 2020). Evidence suggests that probiotics can positively affect bone metabolism and overall skeletal integrity through various mechanisms (Dong et al., 2024). They have been shown to enhance bone density and strength by stimulating the activity of osteoblasts, the cells responsible for forming new bone. This is particularly advantageous in the management of bone-related conditions such as osteoporosis, where issues of bone loss and fragility are prevalent (Xu et al., 2023). Furthermore, probiotics help in reducing long-term inflammation, which is usually linked to bone diseases like arthritis and osteoporosis, by adjusting the immune system (Collins et al., 2017). Additionally, they aid in better gut health and the absorption of nutrients, making sure important nutrients for bone health, like calcium and vitamin D, are properly used. Bone tissue, recognized as a mineralized connective tissue, plays an essential role in numerous physiological processes. However, its integrity can be compromised due to fractures or defects resulting from surgical interventions, trauma, or the excision of tumors (Bauso et al., 2024). While traditional treatment options such as autografts and allografts are effective, they are associated with certain risks, including the potential for disease transmission, chronic pain, infection, immunogenic responses, and limitations in availability. In this context, nanomaterials emerge as a promising alternative, demonstrating superior efficacy in facilitating cell adhesion, proliferation, and subsequent bone regeneration compared to traditional micro-sized materials (Wang et al., 2022). Specifically, nanoparticles that closely resemble the size of natural bone constituents, such as hydroxyapatite crystals, hold considerable promise for localized applications in bone repair. It is possible to create modular systems that allow for the spatial and temporal regulation of the release of physiologically active substances (Mitchell et al., 2014). Furthermore, nanoparticles can be applied as coatings on the surfaces of implants or employed for transmembrane transport in fields such as cell labelling and gene therapy (Liang et al., 2024). The development of modular systems is feasible, enabling the controlled release of physiologically active substances in both spatial and temporal contexts (Eleftheriadis et al., 2021). Alternatively, nanoparticles can be utilized as coatings on implant surfaces or for transmembrane transport in applications like cell labelling and gene therapy (Turnbull et al., 2018). The localized application of nanoparticles has the potential to enhance tissue regeneration, improve the osseointegration of implants, and mitigate the risk of infections. Nanomaterials play a significant role in bone healing through several essential mechanisms. They enhance osteogenesis by facilitating the proliferation and differentiation of osteoblasts and stem cells into osteogenic lineages (Wang and Yeung, 2017). By creating surfaces or coatings that replicate the extracellular matrix (ECM), they establish an environment that promotes cell attachment and growth. Additionally, engineered nanomaterials can transport bioactive molecules, such as growth factors and signalling peptides, directly to the site of the defect, thereby further aiding in osteoblast differentiation and bone formation (Aazmi et al., 2024). In addition, nanomaterials contribute mechanical support by establishing porous scaffolds that possess large surface areas and precisely controlled pore sizes, thereby facilitating cell infiltration and the regeneration of new bone tissue (Golebiowska et al., 2024). These scaffolds can be designed to replicate the stiffness and elasticity of natural bone, including its stiffness and elasticity, thus providing necessary support during the healing process. The bioactivity of these materials is further augmented through surface functionalization, such as the application of hydroxyapatite coatings, which facilitates new bone formation (Morwood et al., 2023). Nanomaterials possess significant anti-inflammatory and antibacterial characteristics that are essential for effective bone healing (Dediu et al., 2023). They can diminish inflammation at the site of injury and mitigate the risk of infections, a factor of utmost importance in clinical environments where infections may hinder the healing process (Zhang et al., 2024a). Moreover, nanomaterials facilitate controlled release systems that enhance therapeutic interventions by consistently delivering drugs or growth factors, ensuring that bone repair receives continuous and effective support (Egwu et al., 2024). Ultimately, nanomaterials can facilitate angiogenesis, the creation of new blood vessels, by providing angiogenic factors or fostering blood vessel development within the scaffold (Krishani et al., 2023). This process is vital for enhancing the delivery of nutrients and oxygen to the healing bone, which is essential for successful repair and regeneration. To summarize, the various ways in which nanomaterials impact bone healing underscore their potential as cutting-edge solutions for addressing bone defects and enhancing recovery outcomes (Han et al., 2023). This novel approach not only overcomes the limitations associated with conventional treatments but also represents a substantial progression in the fields of orthopaedic and regenerative medicine.An important development in the treatment of bone disorders is the new use of probiotics-beneficial bacteria that improve gut health and regulate immune responses in conjunction with nanomaterials (Kerry et al., 2018). Research has demonstrated that probiotics contribute to bone health by increasing bone density, promoting osteoblast activity, and reducing inflammation (Parvaneh et al., 2014). Probiotics and nanomaterials combined strategically may offer a complete approach to improving bone health and quickening the healing process. The combination of probiotics and nanomaterials represents a novel approach that combines the benefits of microbial therapies with the cutting-edge biomaterials’ advantages (Sun et al., 2023a). By using nanomaterials to provide targeted support for bone regeneration and repair and probiotics to create a healthier systemic environment that improves bone health and reduces inflammation, this synergistic approach aims to increase the effectiveness of treatments for bone disorders (Jampilek and Placha, 2021). The integration of these technologies offers a more comprehensive approach to treating bone disorders, which may improve patient outcomes and hasten recovery (Chindamo et al., 2020). By addressing the complex issues surrounding bone health from multiple angles, this integrated approach represents a significant advancement in the domains of orthopaedic and regenerative medicine (Abd-Elkawi et al., 2023). **Table 1** presents a summary of the impact of different probiotic strains and nanoparticles on various bone marrow diseases, such as osteoarthritis, osteoporosis, bone defects, and osteosarcoma. It emphasizes that probiotics like *Lactobacillus rhamnosus, Lactobacillus casei, Bifidobacterium longum*, and *Saccharomyces boulardii* have been investigated for their potential to enhance hematopoiesis, decrease inflammation, and improve overall patient outcomes. Reported effects include higher blood cell counts, decreased chemotherapy side effects, and better gut health.

**Table 1:** Impact of probiotics and nanoparticles on bone marrow diseases.

Bone marrow diseases	Probiotic strain / Nanoparticles studied	Reported Effects	Model
Bone defect (Guo et al., 2022)	Calcium carbonate NPs, silver NPs	Improved osteogenic and osteoinductive properties	Rabbit
Osteoporosis (Dwivedi et al., 2015)	*Lactobacillus casei*	Promoted fracture healing	Mice
Osteoarthritis (Zhang et al., 2016)	Gold NPs	Stimulates chondrocyte proliferation and enhances extracellular matrix production	Cell lines
Osteoarthritis (Amdekar et al., 2011)	*Lactobacillus casei*	Decreased the secretion of Pro-inflammatory cytokines (TNF-α and IL-6)	Wistar rats
Osteoarthritis (Korotkyi et al., 2021)	probiotic composition (PB) and chondroitin sulfate (CS)	Enhancing the anti-inflammatory and antioxidant effect	Rats
Osteoporosis (Gholami et al., 2022)	*Lactobacillus acidophilus, Limoslactobacillus reuteri, Lacticaseibacillus casei, Bifidobacterium longum, and Bacillus coagulans*	Improved bone formation, reduced bone resorption	Dawley rats
Osteoporosis (Dar et al., 2018)	*Lactobacillus Acidophilus*	Decreased NLRP3 inflammasome	Rats
Osteoporosis (Resciniti et al., 2024)	*Lactobacilli*-Based Probiotic Food	Increased bone mineral density	Postmenopausal Women
Osteoporosis (Marycz et al., 2021)	Iron Oxide NPs	Regeneration of osteoporotic bone and inhibit the growth of osteoclast	*In Vitro*
Bone defect (Paltanea et al., 2023)	Magnetic NPs	Low inflammation	Rats
Osteoporosis (Marycz et al., 2020b)	Fe3O4 NPs	Inhibit Osteoclastogenesis, Anti-inflammatory effect	*In-Vitro*
Osteoporosis (Marycz et al., 2020a)	α-Fe2O3/γ-Fe2O3 NPs	Reduce inflammation and enhance osteogenic differentiation of osteoblast	*In-Vitro*
Osteosarcoma (Li et al., 2019a)	Magnetic NPs	Good effect in bone repair,	Rabbit
Osteosarcoma (Gong et al., 2020)	Polyethylenimine-dextran-coated magnetic NPs	Enhancing the anti-OS effect of miR302b and low cytotoxicity	Nide Mice
Osteoporosis (Fouad-Elhady et al., 2020)	Nanohydroxyapatite, chitosan/hydroxyapatite nanocomposites and silver/hydroxyapatite NPs	Effectiveness of NHA, NCH/HA, and NAG/HA as possible anti-resorptive nano biomaterials for treating primary osteoporosis	Wistar rats

## Bone biology

**Figure 1:**
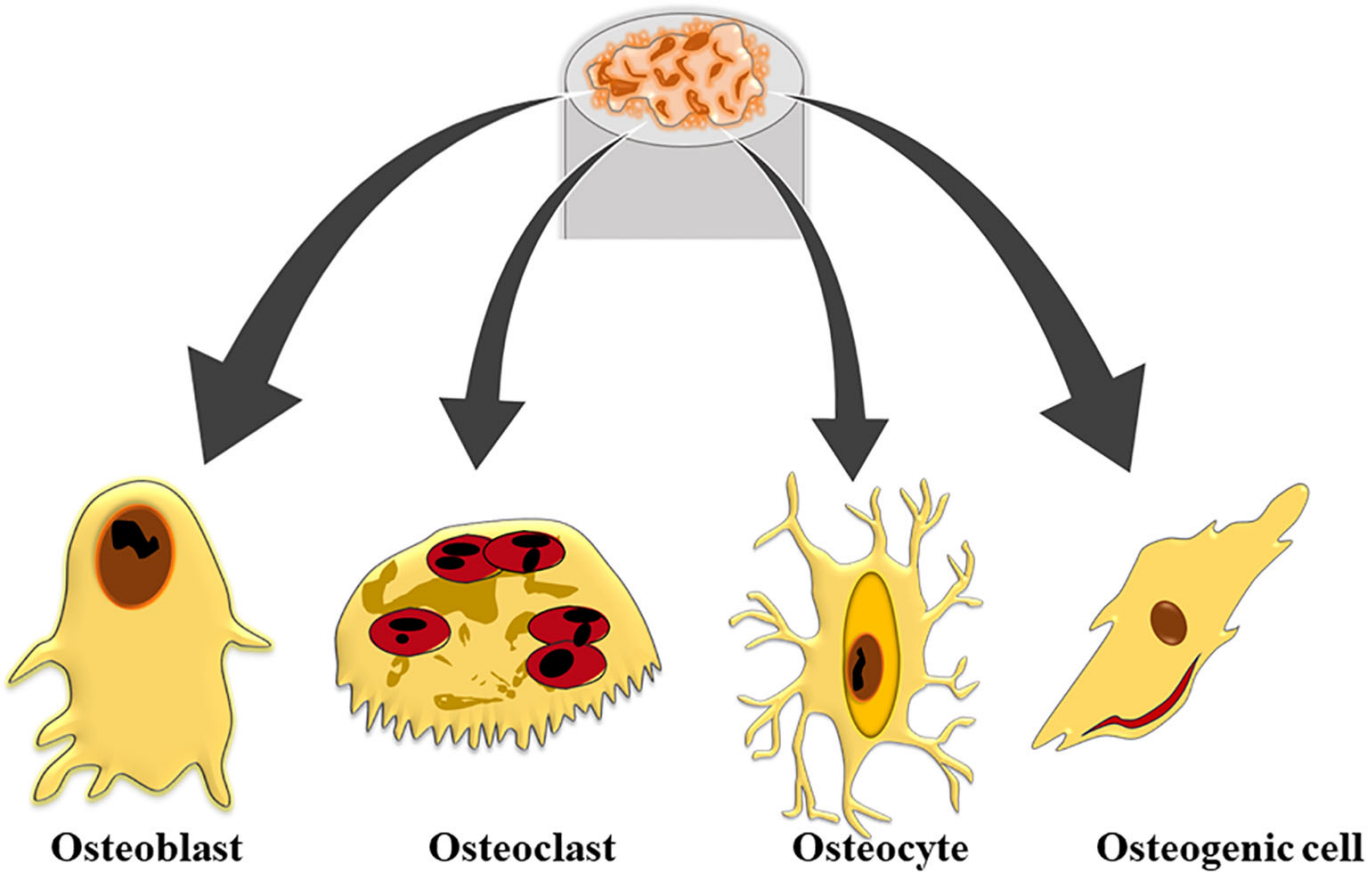
Schematic representation of the types of bone cells.

A key component of bones, bone marrow is located inside the bone cavities and is responsible for producing and controlling the number of blood cells. Bone marrow, which is mostly found in the pelvis, ribs, sternum, and vertebrae, allows for the production and release of blood cells, a process known as hematopoiesis. Bone is a rigid tissue that supports and protects the body’s organs while providing a complex structure essential for overall function (Boes and Durham, 2017). Composed of both inorganic and organic materials, such as hydroxyapatite and collagen fibers, bone’s unique mechanical characteristics and porosity arise from the interaction between this components (Eriksen, 2010). The collagen fibres impart flexibility and strength, while hydroxyapatite, a form of calcium phosphate, adds rigidity and density. This combination creates the ordered, resilient structure of the skeletal system. Bones are not static; they are continuously remodelled through the work of osteoblasts, which build new bone, and osteoclasts, which resorb old bone (Florencio-Silva et al., 2015). This dynamic process is crucial for maintaining bone health, adapting to mechanical stress, and repairing micro-damages. Additionally, bones act as reservoirs for vital minerals like calcium and phosphorus, which are necessary for functions such as nerve transmission and muscle contraction (Ansari, 2019). When bones are compromised or distorted, the integrated structure and function of the skeletal system are disrupted, potentially impacting overall quality of life. Understanding bone biology is essential for effectively addressing conditions such as osteoporosis, fractures, and other skeletal disorders (Scheinpflug et al., 2023). Bone tissue is categorized into two primary histological types: compact bone and spongy bone. Compact bone, or cortical bone, is dense and forms the outer layer of bones, providing strength and rigidity through its tightly packed structural units called osteons or Haversian systems. These osteons consist of concentric lamellae, which are layers of collagen fibers embedded in a mineralized matrix, arranged around a central canal containing blood vessels and nerves (Defranoux et al., 2005). In contrast, spongy bone, or trabecular bone, features a porous, lattice-like structure made up of thin bony plates called trabeculae. This type of bone is found primarily at the ends of long bones and within the interior of others, offering structural support and flexibility while reducing weight (Sykes, 1992). Spongy bone also houses red marrow, which is essential for hematopoiesis and contains pluripotent mesenchymal stem cells (MSCs) capable of differentiating into various tissues including bone, cartilage, muscle, and adipose tissue. Both compact and spongy bone play crucial roles in the overall function and health of the skeletal system (Hart et al., 2020). The orientation of trabecular networks in spongy bone reflects the optimal directional transmission of force and contributes significantly to bone remodeling and healing processes. Additionally, bone tissue includes four cell types-osteoblasts, osteocytes, osteoclasts, and osteogenic cells that are embedded within the extracellular bone matrix, each contributing to the maintenance and remodeling of bone (**Figure 1**).

## Bone marrow disorders

### Leukemia

The unchecked growth of aberrant white blood cells is the hallmark of leukemia, a malignancy that develops in the bone marrow. Fatigue, frequent infections, and easy bruising or bleeding are symptoms caused by these malignant cells that crowd out normal blood cells (Kaspers and Creutzig, 2005). Several subtypes of leukemia exist, including acute lymphoblastic leukemia (ALL), acute myeloid leukemia (AML), chronic lymphocytic leukemia (CLL), and chronic myeloid leukemia (CML), each requiring distinct treatment approaches. While ALL can occur in adults, it is more prevalent in children and is characterized by the rapid proliferation of immature lymphocytes, a type of white blood cell (Wu et al., 2021). Intense chemotherapy is a common part of treating ALL, and stem cell transplantation is an option for some patients. In Acute Myeloid Leukemia (AML), a buildup of immature myeloid cells in the bone marrow and circulation is caused by an attack on the myeloid lineage of blood cells. Adult-onset AML typically necessitates intensive chemotherapy, with stem cell transplantation frequently following (Coustan-Smith et al., 2000). CLL, or Chronic Lymphocytic Leukemia, is a slower-growing form of leukemia that mostly strikes the elderly. The condition is marked by an overabundance of aberrant lymphocytes in various lymphoid organs, blood, and bone marrow (Pedersen-Bjergaard et al., 1981). Chemotherapy, targeted medicines, and stem cell transplantation are all potential treatments for chronic lymphocytic leukemia. In chronic myeloid leukemia (CML), a genetic mutation called the Philadelphia chromosome causes myeloid cells to multiply uncontrollably. In order to combat the aberrant protein that the Philadelphia chromosome produces, tyrosine kinase inhibitors (TKIs) form the backbone of CML treatment (Gonçalves et al., 2010).

### Hematologic malignancy

Lymphoma is a malignancy that affects the lymphatic system, which comprises the bone marrow, thymus, spleen, and lymph nodes. Nothernia lymphoma (NHL) and Hodgkin lymphoma (HL) are the two primary categories into which lymphomas are typically placed (Eneroth and Moberger, 1973). Both kinds can impact the generation of blood cells by interacting with the bone marrow. Reed-Sternberg cells are a hallmark of Hodgkin lymphoma (HL), an aberrant cell type prevalent in lymphoid organs (Qiu et al., 2021). When HL reaches the bone marrow, it can cause abnormalities in the cells that make the blood, such as anemia. Chemotherapy and radiation therapy are the mainstays of HL treatment, with stem cell transplantation reserved for patients who do not respond to these methods (Shanbhag and Ambinder, 2018). Non-Hodgkin Lymphoma (NHL): Numerous lymphomas with lymphatic system origins fall under the umbrella of NHL. The bone marrow can be affected by some subtypes of NHL, including diffuse large B-cell lymphoma (DLBCL) and follicular lymphoma. Depending on the subtype and stage of the disease, treatment options for NHL might range from radiation therapy to stem cell transplantation, chemotherapy, targeted treatments, and targeted therapies (Batlevi and Younes, 2013).

### Anemia of apoplexy

An extremely rare and life-threatening disorder, aplastic anemia occurs when the bone marrow does not make enough of every kind of blood cell (pancytopenia). Anemia, heightened infection risk, and excessive bleeding are all outcomes of this failure (Falhammar et al., 2021). Autoimmune illnesses, hazardous chemical exposure, certain drugs, and viral infections are among the many potential causes of aplastic anemia. Depending on the severity of the condition, immunosuppressive therapy, stem cell transplantation, and blood transfusions may be used to treat aplastic anemia (Paulson, 1971). Antithymocyte globulin (ATG) and cyclosporine are commonly used immunosuppressive agents for treating diseases driven by autoimmune processes Myelodysplastic syndromes (MDS) occur when the bone marrow fails to generate healthy blood cells effectively. This condition can lead to cytopenias, including anemia, neutropenia, and thrombocytopenia, which result from impaired hematopoiesis and dysfunctional myeloid cell regulation. The cytopenia’s that can develop as a result of myeloid-derived suppressive syndrome include anemia, neutropenia, and thrombocytopenia. Acute myeloid leukemia (AML) can develop in some instances, while the disease can manifest in a wide spectrum of severity (Giammattei et al., 2016). Blood transfusions and growth factor therapy are examples of supportive care for myelodysplastic syndromes (MDS), while lenalidomide and hypomethylating drugs (e.g., azacitidine) are examples of disease-modifying medicines. Patients with high-risk MDS, especially those who are younger, may be candidates for stem cell transplantation in certain situations (Scheidegger, 1955).

## Current diseases

A wide range of bone marrow disorders can impair the body’s ability to produce and regulate blood cells effectively. **Table 2** outlines the associated symptoms and complications that arise when these conditions affect different components of the hematological system. To ensure clarity and consistency, we have expanded the discussion to include detailed explanations of the most common bone marrow diseases, their distinguishing features, and the current diagnostic methods used to identify them.

**Table 2:** Overview of major bone marrow diseases.

Disease	Affected Blood Cells	Symptoms	Diagnostic Methods	Current Treatments
Leukemia (Hallek, 2019)	White Blood Cells	Fatigue, frequent infections, easy bruising, bleeding, bone pain	Blood tests, bone marrow biopsy, cytogenetic analysis	Chemotherapy, radiation, stem cell transplant
Lymphoma (Winkler et al., 1999)	Lymphocytes	Swollen lymph nodes, night sweats, weight loss, fatigue, fever	Lymph node biopsy, imaging tests, blood tests	Chemotherapy, radiation, immunotherapy, targeted therapy
Aplastic Anemia (Miano and Dufour, 2015)	All Blood Cells	Fatigue, shortness of breath, pale skin, frequent infections, bleeding	Blood tests, bone marrow biopsy	Blood transfusions, immunosuppressive therapy, stem cell transplant
Myelodysplastic Syndromes (MDS) (Gangat et al., 2016)	All Blood Cells	Fatigue, shortness of breath, bleeding, frequent infections	Blood tests, bone marrow biopsy, cytogenetic analysis	Supportive care, chemotherapy, stem cell transplant
Multiple Myeloma (Rajkumar and Kumar, 2020)	Plasma Cells	Bone pain, fractures, fatigue, kidney dysfunction, frequent infections	Blood tests, urine tests, bone marrow biopsy, imaging tests	Chemotherapy, immunotherapy, targeted therapy, stem cell transplant
Thalassemia (Ali et al., 2021)	Red Blood Cells	Fatigue, weakness, pale or yellowish skin, facial bone deformities	Blood tests, genetic testing	Blood transfusions, iron chelation therapy, stem cell transplant
Sickle Cell Disease (Pinto et al., 2019)	Red Blood Cells	Pain episodes, fatigue, swelling, frequent infections, delayed growth	Blood tests, genetic testing	Pain management, blood transfusions, stem cell transplant

## Emerging role of nanotechnology and probiotics in bone marrow disorders

### Biomaterials

Biomaterials are substances designed to interact with biological systems for medical purposes, such as diagnosing, treating, or replacing tissues and organs (Gao et al., 2017). These materials can be natural, synthetic, or a combination of both, and are engineered to perform specific functions like supporting tissue regeneration, providing structural support, or delivering therapeutic agents (Nguyen et al., 2024). For a biomaterial to be effective, it must exhibit several critical characteristics: biocompatibility to prevent adverse reactions, nontoxicity to ensure safety, appropriate mechanical and physical properties to function properly, rust and corrosion resistance to avoid degradation, and the ability to be designed and manufactured into various forms affordably (Bharadwaz and Jayasuriya, 2020). Biomaterials are categorized into several types based on their composition and applications. Metal biomaterials, such as cobalt-based alloys, titanium, and stainless steel, are commonly used for implants due to their durability. Polymer biomaterials, including materials like polyethylene, polyurethane, and polylactic acid, offer good elastic and plastic properties and are used in various medical devices (Fan et al., 2024). Composite biomaterials, combining different materials to achieve low weight and high strength, are often used in prosthetics. Ceramic biomaterials, known for their hardness and strength, are widely used in dentistry for dentures and dental cements. Natural biomaterials, derived from living organisms like collagen, are advantageous due to their similarity to body tissues and their ability to aid in tissue healing (Battafarano et al., 2021). However, natural biomaterials can face issues such as immunogenicity, degradation, and limited fabrication processes (Alshangiti et al., 2023). In addition to these engineered biomaterials, autografts, allografts, and xenografts, which are transplant materials derived from the patient, a donor, or another species, respectively, are also used in medical treatments. Despite their versatility, biomaterials often face challenges like limited functionality, potential toxicity, and foreign body responses, which restrict their applications and effectiveness (de Paula et al., 2023). Biomaterials aid in the treatment of bone marrow diseases through several key mechanisms. They provide essential support by creating scaffolds that mimic the natural extracellular matrix, facilitating the regeneration of healthy bone marrow tissue (Kowalczewski and Saul, 2018). Biomaterials can also deliver stem cells directly to the affected area, enhancing the restoration of functional bone marrow. Additionally, they enable controlled release of therapeutic agents, such as growth factors or chemotherapy drugs, to target specific disease aspects while minimizing side effects (Dec et al., 2022). Some biomaterials possess immune-modulating properties that can reduce inflammation and immune-mediated damage, crucial for diseases involving immune dysfunction. They may also have hemostatic properties that help manage bleeding disorders associated with bone marrow failure (Cheng et al., 2022). By supporting tissue engineering and regenerative approaches, biomaterials contribute to the effective management and treatment of bone marrow diseases, ultimately improving patient outcomes (Park et al., 2024).

### Nanoparticles as adjuvant therapy in bone marrow diseases

**Figure 2:**
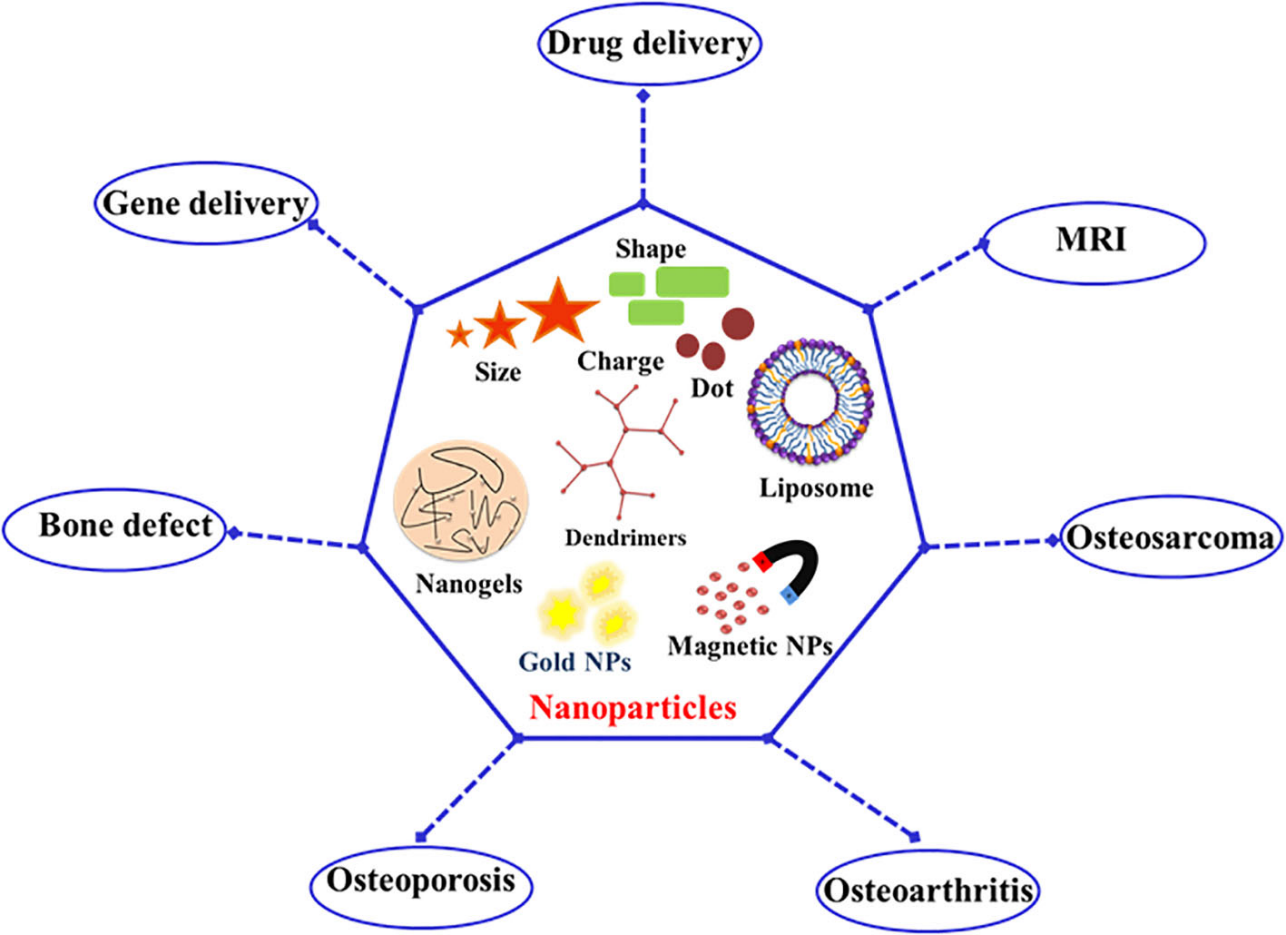
Applications of nanoparticles in bone regeneration and disease management.

Nanoparticles are transforming bone regeneration through their unique properties and applications, leveraging their high surface area-to-volume ratio and customizable surface chemistry (Yang et al., 2024). They enhance bone healing by precisely delivering growth factors, drugs, or genes to targeted areas, thereby promoting bone formation and accelerating repair processes (Shi et al., 2024). Nanoparticles are also integrated into scaffolds or hydrogels to improve their mechanical properties and biocompatibility, while supporting cell adhesion and proliferation. By stimulating osteoblast activity or inhibiting osteoclast activity, nanoparticles influence bone remodeling and enhance the quality of regenerated bone (Wang et al., 2023). Their interaction with biological systems at the molecular level enables advanced therapies, making them crucial in regenerative medicine (Berthiaume et al., 2011). Recently, nanoparticles have gained attention as versatile drug delivery vehicles due to their superior pharmacokinetic properties, sustained release capabilities, and targeted delivery to specific cells or tissues (Huang et al., 2024). This has significantly enhanced the efficacy of existing drugs through targeted delivery and the enhanced permeability and retention effect (Rosenblum et al., 2018). Nanoparticles have proven effective in treating skeletal-related diseases, such as osteoporosis, osteoarthritis, osteosarcoma, and bone defects or repairs, and are extensively used in bone tissue engineering for drug and gene delivery, as well as cell labeling and MRI (**Figure 2**). These applications improve treatment efficiency, enable accurate *in vivo* cell tracking, and enhance diagnostic capabilities, thereby advancing both therapeutic and preventive strategies in bone disease management (Rosenblum et al., 2018).

### History and uses of microorganisms as probiotics

The use of probiotics in the treatment of bone marrow diseases has a historical basis and growing scientific interest. Probiotics have a long history, dating back to ancient times, when fermented foods were consumed for their health benefits (Vanitchanont et al., 2024). However, in the early twentieth century, Elie Metchnikoff, a Russian scientist, proposed that the lactic acid bacteria contained in yoghurt may increase health and life (Gul and Durante-Mangoni, 2024). This early concept attributes the foundation for modern probiotic research, which has developed significantly in recent decades to investigate their potential benefits for a variety of health conditions, including bone marrow disorders (Latif et al., 2023). Probiotics are known for their ability to modulate the immune system, an important factor in the treatment of bone marrow disorders and characterized by immune dysfunction. By enhancing immune function and reducing inflammation, probiotics have the potential to improve diseases such as leukemia and aplastic anemia. Studies have shown that probiotics can impact cytokine production and immune cell function, which shows promise in the management of these diseases (Mazziotta et al., 2023). Furthermore, the interlink between the gut health and bone marrow function, known as the gut-bone marrow axis, implies that maintaining a healthy gut microbiome with probiotics may positively influence bone marrow health and overall immune response (Inchingolo et al., 2024). Probiotics may provide supportive advantages for individuals undergoing treatments for bone marrow diseases, such as chemotherapy, which can disrupt the gut microbiome and immune system function (Hashemi et al., 2023). Probiotics have been investigated as a potential remedy for digestive issues. The use of probiotics has been demonstrated by clinical trials to decrease the frequency of diarrhea resulting from chemotherapy and enhance the overall quality of life for cancer patient (Zhang et al., 2024c).

### Mechanism action of probiotics for bone marrow diseases

Probiotics have an influence on bone marrow illnesses through a number of pathways, including immunological regulation, gastrointestinal health, and inflammation (Cristofori et al., 2021). Probiotics can benefit people with bone marrow problems primarily by modulating their immune systems. Probiotic strains can boost anti-inflammatory cytokines like IL-10 and decrease pro-inflammatory cytokines like TNF-α by improving immune cell function and cytokine production (Azad et al., 2018). This can help manage immunological dysregulation caused by illnesses such as leukemia and aplastic anemia (Airola et al., 2023). An additional crucial mechanism to consider is the gut-bone marrow axis, highlighting the significance of gut health in relation to bone marrow function. Probiotics play a role in maintaining a healthy balance of gut flora, impacting systemic inflammation and immune responses. They support the production of short-chain fatty acids (SCFAs), like butyrate, known for their anti-inflammatory properties that can enhance bone marrow health (Ryma et al., 2021). Imbalances in gut microbiota can lead to systemic inflammation, negatively affecting bone marrow, hence probiotics may assist in promoting a beneficial gut flora (Di Vincenzo et al., 2024). Probiotics play a crucial role in maintaining the integrity of the intestinal barrier. By enhancing the synthesis of tight junction proteins and reducing intestinal permeability, they offer significant benefits, particularly in situations such as chemotherapy where gut barrier function may be compromised, resulting in systemic inflammation and secondary infections (DiMattia et al., 2024). The use of probiotics can potentially mitigate these adverse effects, promoting overall well-being and supporting individuals with bone marrow disorders (Maftei et al., 2024). Furthermore, probiotics have anti-inflammatory characteristics, which may improve bone marrow function. They can reduce systemic inflammation by controlling inflammatory mediator synthesis and immune cell activity. Certain strains, such as *Lactobacillus* and *Bifidobacterium*, have been shown to lower levels of inflammatory markers such as C-reactive protein (CRP) and interleukin-6 (IL-6), which are typically increased in bone marrow disorders (Virk et al., 2024). This reduction in inflammation may be useful in the treatment of chronic inflammation associated with certain disorders. The use of probiotics in cancer therapies, such as chemotherapy, could potentially bring about therapeutic advantages. Studies have indicated that probiotics may play a role in reducing side effects like diarrhea and mucositis, which can have implications for treatment outcomes and the well-being of patients (Mego et al., 2013). Through the mitigation of gastrointestinal symptoms and the promotion of gut health, probiotics may improve treatment tolerance and overall health, thus positively impacting bone marrow health.

### Recent advances and new trends

Recent advances in the use of probiotics to treat bone marrow diseases have provided vital insights into their therapeutic potential. One significant development is a better understanding of the immune system’s effect on probiotics, which is critical for managing bone marrow illnesses (Montazeri-Najafabady et al., 2022). Probiotic strains can improve immune responses by increasing anti-inflammatory cytokines like IL-10 and decreasing pro-inflammatory cytokines like TNF-α. This immune modulation may be especially useful in treating illnesses such as leukemia and aplastic anemia, where immunological dysregulation is a major problem (Raheem et al., 2021). The gut-bone marrow axis has received attention in recent study, emphasizing the importance of gut health in bone marrow function. Probiotics are required to maintain a healthy gut flora, which influences systemic inflammation and immunological responses (Zhang et al., 2024d). Recent research has found that probiotics help to produce short-chain fatty acids (SCFAs), such as butyrate, which have anti-inflammatory effects and can improve bone marrow function. It is now recognized that alterations in the gut microbiota can cause systemic inflammation, impairing bone marrow function, making probiotic intervention a viable therapy (Duan et al., 2023). Progress in microbiome research has considerably improved our understanding of probiotic strain-specific effects. Recent research is focusing on determining which strains have the most impact on bone marrow health (Hitch et al., 2022). For example, particular strains of *Lactobacillus* and *Bifidobacterium* have shown promise in preclinical investigations for modulating inflammatory responses and facilitating bone marrow regeneration. This more complex approach permits the creation of targeted probiotic medicines tailored to the specific needs of particular patients (Li et al., 2019b). The combination of probiotics with conventional therapies is an increasingly popular area of study. Research is delving into the integration of probiotics with treatments like chemotherapy and stem cell transplants to improve overall results (Górska et al., 2019). Preliminary research indicates that probiotics may have the potential to decrease side effects and enhance gut health, leading to better treatment outcomes for individuals with bone marrow diseases (Liu et al., 2021). This combined approach is seen as a promising strategy to enhance the effectiveness of standard treatments. The trend toward personalized probiotic therapy is also emerging, driven by advances in personalized medicine. Progress in microbiome research has greatly enhanced our understanding of probiotic strain-specific effects. Recent research is focusing on determining which strains have the most impact on bone marrow health. For example, particular strains of Lactobacillus and Bifidobacterium have shown promise in preclinical investigations for modulating inflammatory responses and facilitating bone marrow regeneration (Harahap and Suliburska, 2021).This more complex approach permits the creation of targeted probiotic medicines tailored to the specific needs of particular patients. The increasing number of clinical trials and longitudinal studies is providing essential data on the effectiveness of probiotics in the treatment of bone marrow diseases. These studies are evaluating the safety, efficacy, and long-term advantages of probiotics, especially in preventing or reducing chemotherapy-induced gastrointestinal problems. This research is critical for establishing clear recommendations on the use of probiotics in the management of bone marrow (Fedorak and Madsen, 2004).

## Conclusion

A significant breakthrough in regenerative medicine involves the integration of probiotics with nanoparticles for the treatment of bone disorders, offering a comprehensive strategy for healing and regeneration. The unique size-dependent properties of nanoparticles facilitate the targeted delivery of growth factors and therapeutic agents to specific bone tissues, thereby enhancing cellular responses and accelerating the healing process. Concurrently, probiotics contribute to bone health by modulating the gut microbiome, which plays a crucial role in regulating systemic inflammation and nutrient absorption vital for sustaining bone integrity. The combination of advanced nanotechnology and microbiome research presents synergistic effects that could transform the management of bone disorders, leading to improved patient outcomes through more personalized and effective treatment options.
